# Poly[(*μ*-4,4′-bipyridine)(*μ*-naph­tha­lene-1,4-dicarboxyl­ato)iron(II)]

**DOI:** 10.1107/S1600536808042992

**Published:** 2008-12-20

**Authors:** Jan Boeckmann, Inke Jess, Christian Näther

**Affiliations:** a Institut für Anorganische Chemie, Christian-Albrechts-Universität Kiel, Max-Eyth Str. 2, D-24098 Kiel, Germany

## Abstract

The asymmetric unit of the title compound, [Fe(C_12_H_6_O_4_)(C_10_H_8_N_2_)], consists of two independent Fe(II) atoms, two naphthalene-1,4-dicarboxyl­ate anions and two 4,4′-bipyridine ligands. The Fe(II) atoms are each coordinated by four O atoms of the naphthalene-1,4-dicarboxyl­ate anions and two N atoms of the 4,4′-bipyridine ligands within a distorted octa­hedron. Two Fe(II) atoms are bridged *via* the carboxyl­ate groups of two symmetry-related anions into dimers, which are further connected into chains. These chains are linked by additional anions into layers that are finally connected by the 4,4′-bipyridine ligands into a three-dimensional coordination network.

## Related literature

For a related structure, see: Zheng *et al.* (2005 ▶).
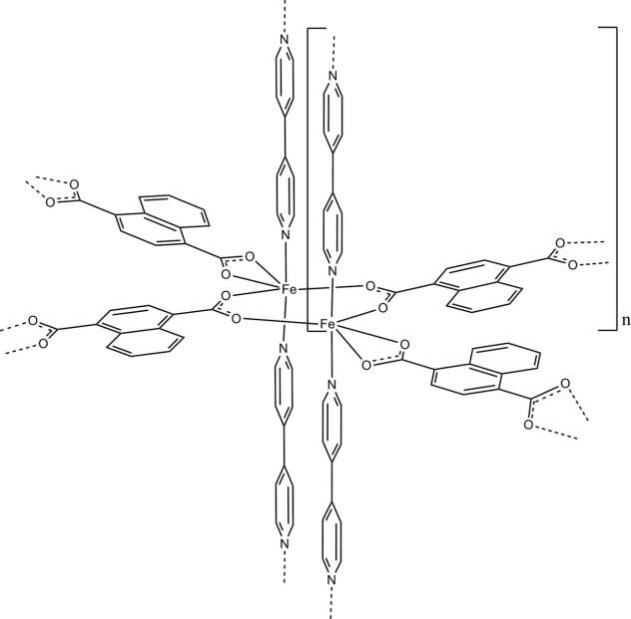


## Experimental

### 

#### Crystal data

[Fe(C_12_H_6_O_4_)(C_10_H_8_N_2_)]
*M* *_r_* = 426.20
Monoclinic, 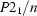

*a* = 10.5169 (4) Å
*b* = 29.8928 (10) Å
*c* = 11.5578 (4) Å
β = 93.178 (3)°
*V* = 3627.9 (2) Å^3^
*Z* = 8
Mo *K*α radiation
μ = 0.87 mm^−1^
*T* = 293 (2) K
0.09 × 0.09 × 0.08 mm

#### Data collection

STOE IPDS-2 diffractometer
Absorption correction: numerical (*X-SHAPE* and *X-RED32*; Stoe, 2008 ▶) *T*_min_ = 0.923, *T*_max_ = 0.933
21152 measured reflections
7116 independent reflections
5305 reflections with *I* > 2σ(*I*)
*R*_int_ = 0.050

#### Refinement

*R*[*F* ^2^ > 2σ(*F* ^2^)] = 0.067
*wR*(*F* ^2^) = 0.141
*S* = 1.14
7116 reflections
523 parameters
H-atom parameters constrained
Δρ_max_ = 0.39 e Å^−3^
Δρ_min_ = −0.36 e Å^−3^

### 

Data collection: *X-AREA* (Stoe, 2008 ▶); cell refinement: *X-AREA*; data reduction: *X-AREA*; program(s) used to solve structure: *SHELXS97* (Sheldrick, 2008 ▶); program(s) used to refine structure: *SHELXL97* (Sheldrick, 2008 ▶); molecular graphics: *DIAMOND* (Brandenburg, 2008 ▶) and *XP* in *SHELXTL* (Sheldrick, 2008 ▶); software used to prepare material for publication: *XCIF* in *SHELXTL*.

## Supplementary Material

Crystal structure: contains datablocks I, global. DOI: 10.1107/S1600536808042992/ng2529sup1.cif

Structure factors: contains datablocks I. DOI: 10.1107/S1600536808042992/ng2529Isup2.hkl

Additional supplementary materials:  crystallographic information; 3D view; checkCIF report

## Figures and Tables

**Table 1:** Selected geometric parameters (Å, °)

Fe1—O4^i^	2.056 (3)
Fe1—O1	2.057 (3)
Fe1—O14	2.215 (3)
Fe1—N2^ii^	2.220 (3)
Fe1—N1	2.227 (4)
Fe1—O13	2.283 (3)
Fe2—O3^i^	2.020 (3)
Fe2—O2	2.048 (3)
Fe2—O11^iii^	2.147 (4)
Fe2—N12^iv^	2.207 (4)
Fe2—N11	2.246 (4)
Fe2—O12^iii^	2.332 (4)

Symmetry codes: (i) 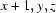; (ii) 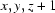; (iii) 
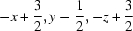
; (iv) 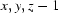.

**Fig. 1.**
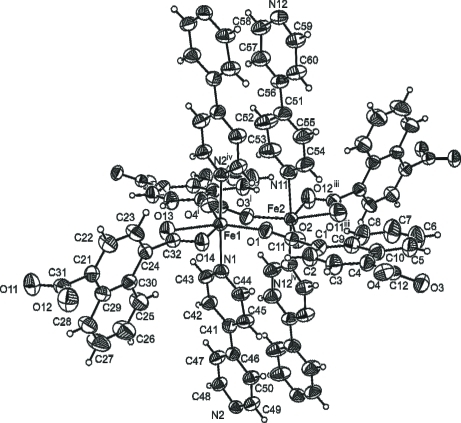
: Crystal structure of the title compund with labelling and displacement ellipsoids drawn at the 50% probability level. Symmetry codes: i = x + 1, y, z; ii = x, y, z + 1; iii = 1.5 - x, -1/2 + y, 1.5 - z; iv = x, y, -1 + z.

**Fig. 2.**
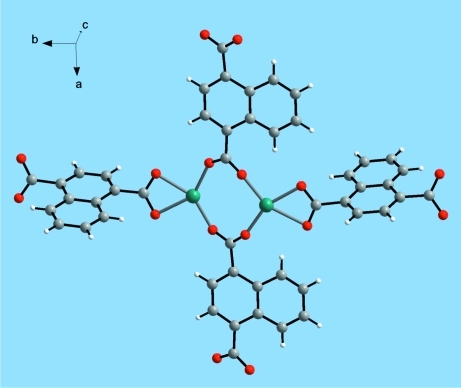
: Crystal structure of the title compound with view of the Fe naphthalene-1,4-dicarboxylate coordination. The co-ligands, 4,4'-bipyridine, are omitted for clarity.

**Fig. 3.**
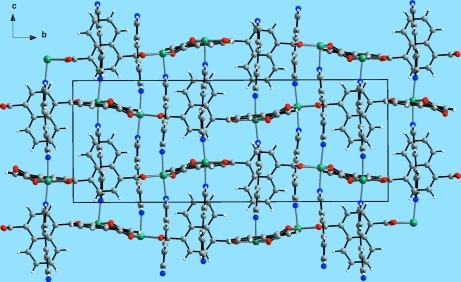
: Crystal structure of the title compound with view in the direction of the crystallographic a axis.

## supplementary crystallographic information

### Comment

The structure determination of the title compound was performed as a part of a project on the synthesis of new metal organic frameworks. In this project we have reacted iron(II)sulfate with naphthalene-1,4-dicarboxylic acid in sodium hydroxide and water, which leads to the formation of bis(µ_2_-4,4'-bipyridine)-bis(µ_2_-naphthalene-1,4-dicarboxylate)diiron(II).

In the crystal structure of the title compound each of the two crystallographically iron atoms are surrounded by two N atoms of two symmetry related 4,4'-bipyridine ligands and four O atoms of three naphthalene-1,4-dicarboxylate anions, of which two are related by symmetry. The coordination polyhedron can be described as a distorted octahedra (Fig 1 and tab 1). Two symmetry related anions bridges two different Fe atoms into dimers, which are further connected into chains by these anions (Fig. 2). Such dimers are also found in the structure of [Eu_2_(naphthalene-1,4-dicarboxylate)_3_(4,4'-bipyridine)_0.5_(H_2_O)_3_]- (4,4'-bipyridine) reported by Zheng *et al.* (2005). The second crystallographically independent napthalene-1,4-dicarboxylate anion is coordinated with both O atoms of its carboxyl group to the metal centers. These anions entangle the Fe-naphthalene-1,4-dicarboxylate chains into layers which are parallel to the a/b plane. These layers are further linked by the 4,4'-bipyridine ligands into a three-dimensional coordination network (Fig 3).

### Experimental

27.9 mg FeSO_4_^.^ 7 H_2_O (0.10 mmol), 33.0 mg naphthalene-1,4-dicarboxylic acid (0.15 mmol), 10.4 mg NaOH (0.26 mmol), 20.0 mg 4,4'-Bipyridine (0.10 mmol) and 5 ml of water were transfered into a glass tube and heated to 150° C for 4 d. On cooling yellow platelets of the title compound were obtained.

### Refinement

All H atoms were located in difference map but were positioned with idealized geometry and were refined isotropic with *U*_eq_(H) = 1.2 *U*_eq_(C) of the parent atom using a riding model with C—H = 0.93 Å.

### Crystal data

**Table:**

[Fe(C_12_H_6_O_4_)(C_10_H_8_N_2_)]	*F*(000) = 1744
*M**_r_* = 426.20	*D*_x_ = 1.561 Mg m^−^^3^
Monoclinic, *P*2_1_/*n*	Mo *K*α radiation, λ = 0.71073 Å
Hall symbol: -P 2yn	Cell parameters from 18988 reflections
*a* = 10.5169 (4) Å	θ = 1.4–27.2°
*b* = 29.8928 (10) Å	µ = 0.87 mm^−^^1^
*c* = 11.5578 (4) Å	*T* = 293 K
β = 93.178 (3)°	Platelets, yellow
*V* = 3627.9 (2) Å^3^	0.09 × 0.09 × 0.08 mm
*Z* = 8	

### Data collection

**Table:**

STOE IPDS-2 diffractometer	7116 independent reflections
Radiation source: fine-focus sealed tube	5305 reflections with *I* > 2σ(*I*)
graphite	*R*_int_ = 0.050
Detector resolution: 0.150 pixels mm^-1^	θ_max_ = 26.0°, θ_min_ = 1.4°
ω scans	*h* = −12→12
Absorption correction: numerical (*X-SHAPE* and *X-RED32*; Stoe, 2008)	*k* = −36→36
*T*_min_ = 0.923, *T*_max_ = 0.933	*l* = −14→12
21152 measured reflections	

### Refinement

**Table:**

Refinement on *F*^2^	Primary atom site location: structure-invariant direct methods
Least-squares matrix: full	Secondary atom site location: difference Fourier map
*R*[*F*^2^ > 2σ(*F*^2^)] = 0.067	Hydrogen site location: inferred from neighbouring sites
*wR*(*F*^2^) = 0.141	H-atom parameters constrained
*S* = 1.14	*w* = 1/[σ^2^(*F*_o_^2^) + (0.0499*P*)^2^ + 3.8737*P*] where *P* = (*F*_o_^2^ + 2*F*_c_^2^)/3
7116 reflections	(Δ/σ)_max_ = 0.001
523 parameters	Δρ_max_ = 0.39 e Å^−^^3^
0 restraints	Δρ_min_ = −0.36 e Å^−^^3^

### Special details

**Table:**

Geometry. All e.s.d.'s (except the e.s.d. in the dihedral angle between two l.s. planes) are estimated using the full covariance matrix. The cell e.s.d.'s are taken into account individually in the estimation of e.s.d.'s in distances, angles and torsion angles; correlations between e.s.d.'s in cell parameters are only used when they are defined by crystal symmetry. An approximate (isotropic) treatment of cell e.s.d.'s is used for estimating e.s.d.'s involving l.s. planes.
Refinement. Refinement of *F*^2^ against ALL reflections. The weighted *R*-factor *wR* and goodness of fit *S* are based on *F*^2^, conventional *R*-factors *R* are based on *F*, with *F* set to zero for negative *F*^2^. The threshold expression of *F*^2^ > σ(*F*^2^) is used only for calculating *R*-factors(gt) *etc*. and is not relevant to the choice of reflections for refinement. *R*-factors based on *F*^2^ are statistically about twice as large as those based on *F*, and *R*- factors based on ALL data will be even larger.

### Fractional atomic coordinates and isotropic or equivalent isotropic displacement parameters (Å^2^)

**Table:**

	*x*	*y*	*z*	*U*_iso_*/*U*_eq_	
Fe1	0.78027 (6)	0.713313 (19)	0.77830 (5)	0.03191 (15)	
Fe2	0.77587 (6)	0.580113 (19)	0.67647 (5)	0.03453 (16)	
C1	0.4126 (4)	0.64573 (15)	0.7149 (4)	0.0431 (10)	
C2	0.3452 (5)	0.68369 (16)	0.7351 (5)	0.0508 (12)	
H2	0.3888	0.7106	0.7453	0.061*	
C3	0.2125 (5)	0.68347 (16)	0.7410 (5)	0.0527 (12)	
H3	0.1709	0.7104	0.7530	0.063*	
C4	0.1427 (4)	0.64543 (15)	0.7297 (4)	0.0408 (10)	
C5	0.1460 (5)	0.56099 (17)	0.7074 (5)	0.0546 (12)	
H5	0.0584	0.5598	0.7143	0.065*	
C6	0.2098 (6)	0.52337 (18)	0.6930 (6)	0.0707 (17)	
H6	0.1658	0.4964	0.6894	0.085*	
C7	0.3436 (6)	0.52322 (18)	0.6828 (6)	0.0742 (18)	
H7	0.3871	0.4966	0.6725	0.089*	
C8	0.4058 (5)	0.56222 (17)	0.6883 (5)	0.0571 (13)	
H8	0.4936	0.5620	0.6817	0.069*	
C9	0.3448 (4)	0.60389 (15)	0.7037 (4)	0.0452 (11)	
C10	0.2088 (5)	0.60334 (15)	0.7128 (4)	0.0447 (10)	
C11	0.5559 (4)	0.64965 (14)	0.7160 (3)	0.0352 (9)	
O1	0.6054 (3)	0.68234 (10)	0.7693 (3)	0.0449 (7)	
O2	0.6173 (3)	0.61999 (11)	0.6670 (3)	0.0469 (8)	
C12	−0.0004 (4)	0.64874 (14)	0.7344 (3)	0.0371 (9)	
O3	−0.0671 (3)	0.61916 (11)	0.6834 (3)	0.0493 (8)	
O4	−0.0452 (3)	0.68212 (11)	0.7840 (3)	0.0458 (7)	
C21	0.7445 (4)	0.94353 (14)	0.8160 (4)	0.0389 (9)	
C22	0.7291 (5)	0.91732 (15)	0.9107 (4)	0.0506 (12)	
H22	0.7117	0.9306	0.9809	0.061*	
C23	0.7392 (5)	0.87040 (16)	0.9035 (4)	0.0498 (12)	
H23	0.7263	0.8531	0.9687	0.060*	
C24	0.7674 (4)	0.84988 (14)	0.8032 (4)	0.0376 (9)	
C25	0.8134 (6)	0.85673 (17)	0.5951 (4)	0.0609 (14)	
H25	0.8218	0.8259	0.5892	0.073*	
C26	0.8274 (7)	0.8826 (2)	0.5001 (5)	0.0779 (19)	
H26	0.8456	0.8692	0.4303	0.093*	
C27	0.8147 (7)	0.9291 (2)	0.5054 (5)	0.0775 (19)	
H27	0.8242	0.9464	0.4395	0.093*	
C28	0.7886 (6)	0.94909 (17)	0.6072 (4)	0.0570 (13)	
H28	0.7802	0.9800	0.6101	0.068*	
C29	0.7740 (4)	0.92362 (15)	0.7085 (4)	0.0426 (10)	
C30	0.7863 (4)	0.87590 (14)	0.7025 (4)	0.0402 (10)	
C31	0.7294 (5)	0.99401 (15)	0.8233 (4)	0.0434 (10)	
O11	0.8268 (4)	1.01802 (11)	0.8276 (3)	0.0608 (9)	
O12	0.6222 (4)	1.01085 (12)	0.8235 (4)	0.0677 (11)	
C32	0.7764 (4)	0.79956 (14)	0.7960 (4)	0.0379 (9)	
O13	0.8823 (3)	0.78071 (10)	0.7884 (3)	0.0496 (8)	
O14	0.6754 (3)	0.77705 (10)	0.7947 (3)	0.0450 (7)	
C41	0.7818 (4)	0.70824 (15)	0.3412 (4)	0.0403 (10)	
C42	0.8945 (4)	0.71142 (17)	0.4109 (4)	0.0454 (11)	
H42	0.9728	0.7109	0.3773	0.054*	
C43	0.8896 (4)	0.71524 (17)	0.5294 (4)	0.0452 (11)	
H43	0.9665	0.7168	0.5731	0.054*	
N1	0.7819 (4)	0.71690 (12)	0.5860 (3)	0.0407 (8)	
C44	0.6740 (4)	0.71451 (16)	0.5187 (4)	0.0447 (10)	
H44	0.5972	0.7159	0.5548	0.054*	
C45	0.6691 (5)	0.71017 (17)	0.3990 (4)	0.0476 (11)	
H45	0.5910	0.7086	0.3574	0.057*	
C46	0.7822 (4)	0.70478 (15)	0.2126 (4)	0.0393 (10)	
C47	0.8938 (4)	0.70362 (16)	0.1559 (4)	0.0458 (11)	
H47	0.9717	0.7026	0.1981	0.055*	
C48	0.8901 (4)	0.70399 (16)	0.0356 (4)	0.0430 (10)	
H48	0.9669	0.7032	−0.0005	0.052*	
N2	0.7827 (3)	0.70538 (11)	−0.0306 (3)	0.0370 (8)	
C49	0.6737 (4)	0.70524 (16)	0.0248 (4)	0.0443 (11)	
H49	0.5970	0.7054	−0.0193	0.053*	
C50	0.6701 (4)	0.70486 (16)	0.1438 (4)	0.0442 (10)	
H50	0.5921	0.7047	0.1780	0.053*	
C51	0.7816 (5)	0.58619 (16)	1.1148 (4)	0.0496 (12)	
C52	0.8930 (6)	0.5839 (2)	1.0576 (5)	0.0728 (17)	
H52	0.9712	0.5846	1.0994	0.087*	
C53	0.8884 (6)	0.5806 (2)	0.9386 (5)	0.0684 (16)	
H53	0.9654	0.5792	0.9029	0.082*	
N11	0.7819 (4)	0.57940 (13)	0.8710 (3)	0.0492 (9)	
C54	0.6749 (6)	0.5807 (2)	0.9274 (4)	0.0596 (14)	
H54	0.5980	0.5794	0.8838	0.071*	
C55	0.6709 (6)	0.5839 (2)	1.0456 (4)	0.0642 (15)	
H55	0.5926	0.5845	1.0793	0.077*	
C56	0.7805 (5)	0.58971 (16)	1.2434 (4)	0.0466 (11)	
C57	0.8913 (5)	0.58902 (19)	1.3130 (4)	0.0573 (13)	
H57	0.9698	0.5894	1.2797	0.069*	
C58	0.8858 (5)	0.58781 (19)	1.4322 (4)	0.0562 (13)	
H58	0.9620	0.5873	1.4771	0.067*	
N12	0.7758 (4)	0.58739 (13)	1.4864 (3)	0.0441 (9)	
C59	0.6696 (5)	0.59193 (17)	1.4197 (4)	0.0512 (12)	
H59	0.5927	0.5945	1.4551	0.061*	
C60	0.6680 (5)	0.59301 (18)	1.3002 (4)	0.0536 (12)	
H60	0.5910	0.5960	1.2574	0.064*	

### Atomic displacement parameters (Å^2^)

**Table:**

	*U*^11^	*U*^22^	*U*^33^	*U*^12^	*U*^13^	*U*^23^
Fe1	0.0347 (3)	0.0338 (3)	0.0273 (3)	−0.0002 (2)	0.0020 (2)	−0.0013 (2)
Fe2	0.0383 (3)	0.0346 (3)	0.0307 (3)	−0.0001 (3)	0.0018 (2)	−0.0012 (2)
C1	0.039 (2)	0.046 (2)	0.044 (2)	0.0056 (19)	0.0007 (19)	−0.006 (2)
C2	0.049 (3)	0.040 (2)	0.063 (3)	−0.004 (2)	0.002 (2)	−0.002 (2)
C3	0.049 (3)	0.043 (3)	0.067 (3)	0.000 (2)	0.008 (2)	−0.004 (2)
C4	0.036 (2)	0.045 (2)	0.042 (2)	−0.0040 (19)	0.0024 (19)	−0.0012 (19)
C5	0.045 (3)	0.055 (3)	0.064 (3)	−0.009 (2)	0.003 (2)	0.000 (2)
C6	0.068 (4)	0.042 (3)	0.101 (5)	−0.006 (3)	0.002 (3)	−0.001 (3)
C7	0.067 (4)	0.038 (3)	0.116 (5)	0.004 (3)	−0.001 (4)	−0.008 (3)
C8	0.039 (3)	0.053 (3)	0.079 (4)	0.001 (2)	0.000 (2)	−0.012 (3)
C9	0.043 (3)	0.044 (2)	0.049 (3)	0.001 (2)	0.002 (2)	−0.001 (2)
C10	0.046 (3)	0.042 (2)	0.046 (3)	−0.002 (2)	0.003 (2)	−0.001 (2)
C11	0.037 (2)	0.037 (2)	0.032 (2)	−0.0013 (18)	0.0017 (17)	0.0018 (17)
O1	0.0465 (18)	0.0490 (18)	0.0393 (17)	−0.0134 (14)	0.0032 (14)	−0.0098 (14)
O2	0.0446 (18)	0.0493 (18)	0.0471 (18)	0.0060 (15)	0.0068 (15)	−0.0029 (15)
C12	0.041 (2)	0.040 (2)	0.030 (2)	0.0008 (19)	0.0008 (17)	0.0007 (17)
O3	0.0446 (18)	0.0543 (19)	0.0489 (19)	−0.0092 (15)	0.0013 (15)	−0.0082 (15)
O4	0.0478 (18)	0.0520 (18)	0.0376 (16)	0.0123 (15)	0.0022 (14)	−0.0081 (14)
C21	0.042 (2)	0.035 (2)	0.040 (2)	−0.0005 (18)	0.0016 (19)	−0.0020 (18)
C22	0.072 (3)	0.039 (2)	0.043 (3)	0.006 (2)	0.016 (2)	−0.002 (2)
C23	0.068 (3)	0.041 (2)	0.041 (3)	0.002 (2)	0.013 (2)	0.007 (2)
C24	0.040 (2)	0.034 (2)	0.039 (2)	−0.0016 (18)	0.0039 (18)	0.0017 (17)
C25	0.098 (4)	0.044 (3)	0.041 (3)	0.000 (3)	0.010 (3)	−0.005 (2)
C26	0.131 (6)	0.064 (4)	0.039 (3)	0.006 (4)	0.016 (3)	−0.004 (3)
C27	0.129 (6)	0.064 (4)	0.041 (3)	0.010 (4)	0.017 (3)	0.010 (3)
C28	0.083 (4)	0.044 (3)	0.045 (3)	0.003 (3)	0.008 (3)	0.006 (2)
C29	0.050 (3)	0.039 (2)	0.038 (2)	−0.001 (2)	−0.001 (2)	0.0025 (18)
C30	0.048 (3)	0.037 (2)	0.035 (2)	−0.0007 (19)	−0.0009 (19)	−0.0019 (18)
C31	0.055 (3)	0.039 (2)	0.035 (2)	0.002 (2)	0.003 (2)	0.0033 (19)
O11	0.067 (2)	0.0405 (18)	0.075 (3)	−0.0081 (17)	0.005 (2)	−0.0023 (17)
O12	0.055 (2)	0.049 (2)	0.098 (3)	0.0128 (17)	0.000 (2)	−0.003 (2)
C32	0.042 (2)	0.038 (2)	0.033 (2)	−0.0023 (19)	−0.0006 (18)	0.0007 (17)
O13	0.0375 (17)	0.0417 (18)	0.070 (2)	0.0018 (14)	0.0034 (15)	−0.0044 (15)
O14	0.0412 (17)	0.0352 (16)	0.059 (2)	−0.0033 (13)	0.0029 (15)	−0.0015 (14)
C41	0.043 (2)	0.045 (2)	0.034 (2)	−0.003 (2)	0.0057 (18)	−0.0001 (18)
C42	0.037 (2)	0.066 (3)	0.034 (2)	−0.004 (2)	0.0033 (18)	0.003 (2)
C43	0.040 (2)	0.065 (3)	0.031 (2)	−0.007 (2)	−0.0013 (18)	0.002 (2)
N1	0.050 (2)	0.0400 (19)	0.0321 (19)	−0.0006 (17)	0.0028 (16)	0.0004 (15)
C44	0.044 (2)	0.062 (3)	0.028 (2)	0.005 (2)	0.0056 (19)	0.002 (2)
C45	0.044 (3)	0.068 (3)	0.031 (2)	0.003 (2)	0.0010 (19)	0.004 (2)
C46	0.041 (2)	0.046 (2)	0.031 (2)	−0.0022 (19)	0.0037 (18)	0.0001 (18)
C47	0.040 (2)	0.063 (3)	0.034 (2)	0.001 (2)	−0.0013 (19)	−0.004 (2)
C48	0.036 (2)	0.060 (3)	0.033 (2)	−0.002 (2)	0.0019 (18)	−0.003 (2)
N2	0.043 (2)	0.0399 (19)	0.0282 (17)	−0.0001 (15)	0.0033 (15)	0.0012 (14)
C49	0.037 (2)	0.062 (3)	0.034 (2)	0.002 (2)	−0.0006 (18)	−0.004 (2)
C50	0.040 (2)	0.060 (3)	0.033 (2)	0.003 (2)	0.0018 (18)	−0.002 (2)
C51	0.068 (3)	0.050 (3)	0.031 (2)	0.006 (2)	0.004 (2)	−0.001 (2)
C52	0.061 (3)	0.121 (5)	0.037 (3)	0.017 (3)	0.000 (2)	−0.005 (3)
C53	0.061 (3)	0.106 (5)	0.038 (3)	0.013 (3)	0.005 (2)	0.004 (3)
N11	0.066 (3)	0.047 (2)	0.035 (2)	0.002 (2)	0.0051 (19)	−0.0004 (17)
C54	0.065 (3)	0.081 (4)	0.032 (2)	−0.010 (3)	−0.004 (2)	−0.001 (2)
C55	0.062 (3)	0.097 (4)	0.034 (3)	−0.005 (3)	0.005 (2)	−0.001 (3)
C56	0.058 (3)	0.051 (3)	0.030 (2)	0.004 (2)	−0.001 (2)	0.0001 (19)
C57	0.059 (3)	0.075 (4)	0.038 (3)	0.001 (3)	0.005 (2)	−0.001 (2)
C58	0.054 (3)	0.081 (4)	0.033 (2)	0.000 (3)	0.002 (2)	−0.006 (2)
N12	0.051 (2)	0.050 (2)	0.0309 (19)	0.0037 (18)	0.0040 (17)	−0.0018 (16)
C59	0.059 (3)	0.062 (3)	0.033 (2)	0.005 (2)	0.003 (2)	−0.006 (2)
C60	0.055 (3)	0.071 (3)	0.034 (2)	0.008 (3)	−0.003 (2)	−0.003 (2)

### Geometric parameters (Å, °)

**Table:**

Fe1—O4^i^	2.056 (3)	C28—C29	1.412 (6)
Fe1—O1	2.057 (3)	C28—H28	0.9300
Fe1—O14	2.215 (3)	C29—C30	1.435 (6)
Fe1—N2^ii^	2.220 (3)	C31—O12	1.235 (6)
Fe1—N1	2.227 (4)	C31—O11	1.249 (6)
Fe1—O13	2.283 (3)	O11—Fe2^vi^	2.147 (4)
Fe2—O3^i^	2.020 (3)	O12—Fe2^vi^	2.332 (4)
Fe2—O2	2.048 (3)	C32—O13	1.256 (5)
Fe2—O11^iii^	2.147 (4)	C32—O14	1.257 (5)
Fe2—N12^iv^	2.207 (4)	C41—C45	1.393 (6)
Fe2—N11	2.246 (4)	C41—C42	1.399 (6)
Fe2—O12^iii^	2.332 (4)	C41—C46	1.490 (6)
C1—C2	1.365 (6)	C42—C43	1.377 (6)
C1—C9	1.442 (6)	C42—H42	0.9300
C1—C11	1.512 (6)	C43—N1	1.340 (6)
C2—C3	1.400 (7)	C43—H43	0.9300
C2—H2	0.9300	N1—C44	1.341 (6)
C3—C4	1.356 (6)	C44—C45	1.388 (6)
C3—H3	0.9300	C44—H44	0.9300
C4—C10	1.456 (6)	C45—H45	0.9300
C4—C12	1.512 (6)	C46—C47	1.377 (6)
C5—C6	1.325 (8)	C46—C50	1.385 (6)
C5—C10	1.428 (7)	C47—C48	1.388 (6)
C5—H5	0.9300	C47—H47	0.9300
C6—C7	1.419 (8)	C48—N2	1.330 (5)
C6—H6	0.9300	C48—H48	0.9300
C7—C8	1.336 (7)	N2—C49	1.344 (5)
C7—H7	0.9300	N2—Fe1^iv^	2.220 (3)
C8—C9	1.417 (7)	C49—C50	1.378 (6)
C8—H8	0.9300	C49—H49	0.9300
C9—C10	1.440 (7)	C50—H50	0.9300
C11—O2	1.250 (5)	C51—C55	1.377 (7)
C11—O1	1.253 (5)	C51—C52	1.379 (7)
C12—O3	1.255 (5)	C51—C56	1.490 (6)
C12—O4	1.256 (5)	C52—C53	1.377 (7)
O3—Fe2^v^	2.020 (3)	C52—H52	0.9300
O4—Fe1^v^	2.056 (3)	C53—N11	1.330 (7)
C21—C22	1.363 (6)	C53—H53	0.9300
C21—C29	1.427 (6)	N11—C54	1.331 (7)
C21—C31	1.520 (6)	C54—C55	1.373 (7)
C22—C23	1.410 (6)	C54—H54	0.9300
C22—H22	0.9300	C55—H55	0.9300
C23—C24	1.358 (6)	C56—C57	1.379 (7)
C23—H23	0.9300	C56—C60	1.387 (7)
C24—C30	1.423 (6)	C57—C58	1.382 (7)
C24—C32	1.510 (6)	C57—H57	0.9300
C25—C26	1.358 (7)	C58—N12	1.346 (6)
C25—C30	1.410 (6)	C58—H58	0.9300
C25—H25	0.9300	N12—C59	1.329 (6)
C26—C27	1.398 (8)	N12—Fe2^ii^	2.207 (4)
C26—H26	0.9300	C59—C60	1.381 (6)
C27—C28	1.361 (8)	C59—H59	0.9300
C27—H27	0.9300	C60—H60	0.9300
			
O4^i^—Fe1—O1	126.27 (13)	C27—C28—C29	121.1 (5)
O4^i^—Fe1—O14	146.62 (12)	C27—C28—H28	119.4
O1—Fe1—O14	86.76 (12)	C29—C28—H28	119.4
O4^i^—Fe1—N2^ii^	87.63 (13)	C28—C29—C21	122.4 (4)
O1—Fe1—N2^ii^	87.88 (13)	C28—C29—C30	118.8 (4)
O14—Fe1—N2^ii^	89.13 (12)	C21—C29—C30	118.8 (4)
O4^i^—Fe1—N1	89.88 (13)	C25—C30—C24	122.8 (4)
O1—Fe1—N1	91.57 (13)	C25—C30—C29	118.0 (4)
O14—Fe1—N1	94.34 (13)	C24—C30—C29	119.1 (4)
N2^ii^—Fe1—N1	176.46 (13)	O12—C31—O11	120.8 (4)
O4^i^—Fe1—O13	89.00 (12)	O12—C31—C21	120.2 (4)
O1—Fe1—O13	144.73 (12)	O11—C31—C21	118.9 (4)
O14—Fe1—O13	58.08 (11)	C31—O11—Fe2^vi^	94.9 (3)
N2^ii^—Fe1—O13	93.68 (13)	C31—O12—Fe2^vi^	86.7 (3)
N1—Fe1—O13	88.78 (13)	O13—C32—O14	120.7 (4)
O3^i^—Fe2—O2	109.11 (13)	O13—C32—C24	120.6 (4)
O3^i^—Fe2—O11^iii^	155.44 (14)	O14—C32—C24	118.6 (4)
O2—Fe2—O11^iii^	95.45 (13)	C32—O13—Fe1	89.1 (2)
O3^i^—Fe2—N12^iv^	86.43 (14)	C32—O14—Fe1	92.1 (3)
O2—Fe2—N12^iv^	86.23 (13)	C45—C41—C42	115.9 (4)
O11^iii^—Fe2—N12^iv^	95.24 (15)	C45—C41—C46	122.0 (4)
O3^i^—Fe2—N11	89.30 (15)	C42—C41—C46	122.0 (4)
O2—Fe2—N11	92.12 (14)	C43—C42—C41	120.1 (4)
O11^iii^—Fe2—N11	89.99 (15)	C43—C42—H42	120.0
N12^iv^—Fe2—N11	174.64 (15)	C41—C42—H42	120.0
O3^i^—Fe2—O12^iii^	97.94 (13)	N1—C43—C42	124.5 (4)
O2—Fe2—O12^iii^	152.87 (13)	N1—C43—H43	117.7
O11^iii^—Fe2—O12^iii^	57.51 (13)	C42—C43—H43	117.7
N12^iv^—Fe2—O12^iii^	93.59 (15)	C43—N1—C44	115.2 (4)
N11—Fe2—O12^iii^	90.21 (15)	C43—N1—Fe1	122.6 (3)
C2—C1—C9	118.5 (4)	C44—N1—Fe1	121.5 (3)
C2—C1—C11	117.5 (4)	N1—C44—C45	124.4 (4)
C9—C1—C11	123.8 (4)	N1—C44—H44	117.8
C1—C2—C3	122.2 (4)	C45—C44—H44	117.8
C1—C2—H2	118.9	C44—C45—C41	119.8 (4)
C3—C2—H2	118.9	C44—C45—H45	120.1
C4—C3—C2	122.3 (5)	C41—C45—H45	120.1
C4—C3—H3	118.8	C47—C46—C50	116.6 (4)
C2—C3—H3	118.8	C47—C46—C41	121.8 (4)
C3—C4—C10	118.5 (4)	C50—C46—C41	121.6 (4)
C3—C4—C12	118.5 (4)	C46—C47—C48	119.9 (4)
C10—C4—C12	123.0 (4)	C46—C47—H47	120.0
C6—C5—C10	121.5 (5)	C48—C47—H47	120.0
C6—C5—H5	119.3	N2—C48—C47	123.6 (4)
C10—C5—H5	119.3	N2—C48—H48	118.2
C5—C6—C7	121.6 (5)	C47—C48—H48	118.2
C5—C6—H6	119.2	C48—N2—C49	116.4 (4)
C7—C6—H6	119.2	C48—N2—Fe1^iv^	122.6 (3)
C8—C7—C6	118.6 (5)	C49—N2—Fe1^iv^	120.8 (3)
C8—C7—H7	120.7	N2—C49—C50	123.2 (4)
C6—C7—H7	120.7	N2—C49—H49	118.4
C7—C8—C9	123.3 (5)	C50—C49—H49	118.4
C7—C8—H8	118.4	C49—C50—C46	120.3 (4)
C9—C8—H8	118.4	C49—C50—H50	119.9
C8—C9—C10	117.3 (4)	C46—C50—H50	119.9
C8—C9—C1	123.2 (4)	C55—C51—C52	115.6 (4)
C10—C9—C1	119.4 (4)	C55—C51—C56	122.0 (5)
C5—C10—C9	117.8 (4)	C52—C51—C56	122.4 (5)
C5—C10—C4	123.3 (4)	C53—C52—C51	119.9 (5)
C9—C10—C4	118.9 (4)	C53—C52—H52	120.0
O2—C11—O1	124.4 (4)	C51—C52—H52	120.0
O2—C11—C1	118.7 (4)	N11—C53—C52	124.8 (5)
O1—C11—C1	116.8 (4)	N11—C53—H53	117.6
C11—O1—Fe1	136.2 (3)	C52—C53—H53	117.6
C11—O2—Fe2	145.8 (3)	C53—N11—C54	114.8 (4)
O3—C12—O4	124.1 (4)	C53—N11—Fe2	124.3 (3)
O3—C12—C4	118.0 (4)	C54—N11—Fe2	120.7 (3)
O4—C12—C4	117.9 (4)	N11—C54—C55	124.2 (5)
C12—O3—Fe2^v^	149.3 (3)	N11—C54—H54	117.9
C12—O4—Fe1^v^	134.6 (3)	C55—C54—H54	117.9
C22—C21—C29	120.0 (4)	C54—C55—C51	120.7 (5)
C22—C21—C31	120.6 (4)	C54—C55—H55	119.7
C29—C21—C31	119.4 (4)	C51—C55—H55	119.7
C21—C22—C23	120.8 (4)	C57—C56—C60	116.1 (4)
C21—C22—H22	119.6	C57—C56—C51	121.8 (5)
C23—C22—H22	119.6	C60—C56—C51	122.1 (4)
C24—C23—C22	121.4 (4)	C56—C57—C58	120.1 (5)
C24—C23—H23	119.3	C56—C57—H57	119.9
C22—C23—H23	119.3	C58—C57—H57	119.9
C23—C24—C30	119.9 (4)	N12—C58—C57	123.3 (5)
C23—C24—C32	120.9 (4)	N12—C58—H58	118.4
C30—C24—C32	119.2 (4)	C57—C58—H58	118.4
C26—C25—C30	121.1 (5)	C59—N12—C58	116.4 (4)
C26—C25—H25	119.5	C59—N12—Fe2^ii^	122.8 (3)
C30—C25—H25	119.5	C58—N12—Fe2^ii^	120.8 (3)
C25—C26—C27	121.1 (5)	N12—C59—C60	123.1 (5)
C25—C26—H26	119.5	N12—C59—H59	118.4
C27—C26—H26	119.5	C60—C59—H59	118.4
C28—C27—C26	119.9 (5)	C59—C60—C56	120.5 (5)
C28—C27—H27	120.0	C59—C60—H60	119.7
C26—C27—H27	120.0	C56—C60—H60	119.7

Symmetry codes: (i) *x*+1, *y*, *z*; (ii) *x*, *y*, *z*+1; (iii) −*x*+3/2, *y*−1/2, −*z*+3/2; (iv) *x*, *y*, *z*−1; (v) *x*−1, *y*, *z*; (vi) −*x*+3/2, *y*+1/2, −*z*+3/2.
